# Systematic Review and Meta-Analysis of Screening Tools for Language Disorder

**DOI:** 10.3389/fped.2022.801220

**Published:** 2022-02-23

**Authors:** Kevin K. H. So, Carol K. S. To

**Affiliations:** Academic Unit of Human Communication, Development, and Information Sciences, Faculty of Education, The University of Hong Kong, Hong Kong, Hong Kong SAR, China

**Keywords:** surveillance, screening, language disorder, PRISMA review, meta-analysis, summary receiver-operating characteristics, meta-regression

## Abstract

Language disorder is one of the most prevalent developmental disorders and is associated with long-term sequelae. However, routine screening is still controversial and is not universally part of early childhood health surveillance. Evidence concerning the detection accuracy, benefits, and harms of screening for language disorders remains inadequate, as shown in a previous review. In October 2020, a systematic review was conducted to investigate the accuracy of available screening tools and the potential sources of variability. A literature search was conducted using CINAHL Plus, ComDisCome, PsycInfo, PsycArticles, ERIC, PubMed, Web of Science, and Scopus. Studies describing, developing, or validating screening tools for language disorder under the age of 6 were included. QUADAS-2 was used to evaluate risk of bias in individual studies. Meta-analyses were performed on the reported accuracy of the screening tools examined. The performance of the screening tools was explored by plotting hierarchical summary receiver operating characteristic (HSROC) curves. The effects of the proxy used in defining language disorders, the test administrators, the screening-diagnosis interval and age of screening on screening accuracy were investigated by meta-regression. Of the 2,366 articles located, 47 studies involving 67 screening tools were included. About one-third of the tests (35.4%) achieved at least fair accuracy, while only a small proportion (13.8%) achieved good accuracy. HSROC curves revealed a remarkable variation in sensitivity and specificity for the three major types of screening, which used the child's actual language ability, clinical markers, and both as the proxy, respectively. None of these three types of screening tools achieved good accuracy. Meta-regression showed that tools using the child's actual language as the proxy demonstrated better sensitivity than that of clinical markers. Tools using long screening-diagnosis intervals had a lower sensitivity than those using short screening-diagnosis intervals. Parent report showed a level of accuracy comparable to that of those administered by trained examiners. Screening tools used under and above 4yo appeared to have similar sensitivity and specificity. In conclusion, there are still gaps between the available screening tools for language disorders and the adoption of these tools in population screening. Future tool development can focus on maximizing accuracy and identifying metrics that are sensitive to the dynamic nature of language development.

**Systematic Review Registration:**

https://www.crd.york.ac.uk/prospero/display_record.php?RecordID=210505, PROSPERO: CRD42020210505.

## Introduction

Language disorder refers to persistent language problems that can negatively affect social and educational aspects of an individual's life (1). It is prevalent and estimated to affect around 7.6% of the population (2). Children with language disorder may experience difficulties in comprehension and/or in the use of expressive languages (3). Persistent developmental language disorder not only has a negative impact on communication but is also associated with disturbance in various areas such as behavioral problems (4), socio-emotional problems (5), and academic underachievement (6).

Early identification of persistent language disorder is challenging. There are substantial variabilities in the trajectories of early language development (7, 8). Some children display consistently low language, some appear to resolve the language difficulties when they grow older, and some demonstrated apparently typical early development but develop late-emerging language disorder. This dynamic nature of early language development has introduced difficulties in the identification process in practice (9). Therefore, rather than a one-off assessment, late talkers under 2 years old are recommended to be reassessed later. Referral to evaluation may not be not based on positive results in universal screening, but mainly concerns from caregivers, the presence of extreme deviation in development, or the manifestation of behavioral or psychiatric disturbances under 5 years old (9). Those who have language problems in the absence of the above conditions are likely to be referred for evaluation after 5 years old. Only then will they usually receive diagnostic assessment.

Ideally, screening should identify at-risk children early enough to provide intervention and avoid or minimize adverse consequences for them, their families, and society, improving the well-being of the children and the health outcomes of the population at a reasonable cost. Despite the high prevalence and big impact of language disorder, universal screening for language disorder is not practiced in every child health surveillance. Screening in the early developmental stages is controversial (10). While early identification has been advocated to support early intervention, there are concerns about the net cost and benefits of these early screening exercises. For example, the US Preventive Task Force reviewed evidence concerning screening for speech and language delay and concluded that there was inadequate evidence regarding the accuracy, benefits, and harms of screening. The Task Force therefore did not support routine screening in asymptomatic children (11). This has raised concerns in the professional community who believe in the benefits of routine screening (12). However, it is undeniable that another contributing factor for the recommendation of the Task Force was that screening tools for language disorder vary greatly in design and construct resulting in the variability in identification accuracy.

Previous reviews of screening tools for early language disorders have shown that these tools make use of different proxies for defining language issues, including a child's actual language ability, clinical markers such as non-word repetition, or both (13). Screening tools have been developed for children at different ages [e.g., toddlers (14) and preschoolers (15)] given the higher stability of language status at a later time point (16, 17). Screening tools also differ in the format of administration. For example, some tools are in the form of a parent-report questionnaire while some have to be administered by trained examiners via direct assessment or observations. Besides the test design, methodological variations have also been noted in primary validation studies, such as the validation sample, the reference standards (i.e., the gold standard for language disorder), and the screening-diagnosis interval. These variations might eventually lead to different levels of screening accuracy, which has been pointed out in previous systematic reviews (10, 13).

These variations have been examined in terms of the screening accuracy (13). Parent-report instruments and trained-examiner screeners have been found to be comparable in screening accuracy. In longitudinal studies in which language disorder status has been validated at various time points, accuracy appears to be lower for longer-term prediction than for concurrent prediction. Although the reviews have provided a comprehensive overview regarding the variations in different language screening tools, the analyses have mainly been based on qualitative and descriptive data. In the current study we performed a systematic review of all currently available screening tools for early language disorders that have been validated against a reference standard. We report on the variations noted in terms of (1) the type of proxy used in defining language disorders, (2) the type of test administrators, (3) the screening-diagnosis intervals and (4) age of screening. Second, we conducted a meta-analysis of the diagnostic accuracy of the screening tools and examined the contributions of the above four factors to accuracy.

## Methods

The protocol for the current systematic review was registered at PROSPERO, an international prospective register of systematic reviews (Registration ID: CRD42020210505, record can be found on https://www.crd.york.ac.uk/prospero/display_record.php?RecordID=210505). Due to COVID-19, the registration was published with basic automatic checks in eligibility by the PROSPERO team. The Preferred Reporting Items for Systematic Reviews and Meta-Analyses for Diagnostic Test Accuracy (PRISMA-DTA) (18) checklist was used as a guide for the reporting of this review.

### Search Strategy

A systematic search of the literature was conducted in 2020 October based on the following databases: CINAHL Plus, ComDisDome, PsycINFO, PsycArticles, ERIC, PubMed, Web of Science, and Scopus. The major search terms were as follows: Child^*^ OR Preschool^*^ AND “Language disorder” ^*^ OR “language impairment^*^” OR “language delay” AND Screening OR identif^*^. To be as exhaustive as possible, the earliest studies available in the databases and those up to October 2020 were retrieved and screened. Appendix A Table A1 showed the detailed search strategies in each database. Articles from the previous reviews were also retrieved.

### Inclusion and Exclusion Criteria

The relevance of the titles, abstracts, and then the full texts were determined for eligibility. Cross-sectional or prospective studies validating screening tools or comparing different screening tools for language disorders were included in the review. The focus was on screening tools validated with children aged 6 or under from the general population or those with referral, regardless of the administration format of the tools, or how language disorder was defined in the studied. Studies that did not report adequate data on the screening results, and in which accuracy data cannot be deduced from the data reported, were excluded from the review (see Appendix A Table A2 for details).

### Data Extraction

Data was extracted by the first author using a standard data extraction form. The principal diagnostic accuracy measures extracted were test sensitivity and specificity. The number of people being true positives (TP), true negatives (TN), false positives (FP) and false negatives (FN) was also extracted. Sensitivity and specificity were calculated based on 2 by 2 contingency tables in the event of discrepancy between the text description and the data reported. The data extraction process was repeated after the first extraction to improve accuracy. Screening tools with both sensitivity and specificity exceeding 0.90 were regarded as good and those with both measures exceeding 0.80 but below 0.90 were regarded as fair (19).

### Quality Assessment

Quality assessment of included articles was conducted by the first author using QUADAS-2 by Whiting, Rutjes (20). QUADAS-2 can assist in assessing risk of bias (ROB) in diagnostic test accuracy studies with signaling questions concerning four major domains. The ROB in patient selection, patient flow, index tests, or the screening tools in the current review, and the reference standard tests were evaluated. Ratings of ROB for individual studies were illustrated using a traffic light plot. A summary ROB figure weighted with sample size was generated using the R package “robvis” (21). Due to the large discrepancy in the sample size across studies, an unweighted summary plot was also generated to show the ROB of the included studies.

### Data Analysis

The overall accuracy of the tools was compared using descriptive statistics. Because sensitivity and specificity are correlated, increasing either one of them by varying the cut-off of test positivity would usually result in a decrease in the other. Therefore, a bivariate approach was used to jointly model sensitivity and specificity (22) in generating hierarchical summary receiver-operating characteristic (HSROC) curves to assess the overall accuracy of screening by proxy and by screening-diagnosis intervals. HSROC is a more robust method accounting for both within and between study variabilities (23).

Three factors that could be associated with screening accuracy, chosen *a priori*, were included in the meta-analysis: proxy used, test administrators, and screening-diagnosis interval. Effect of screening age on accuracy was also evaluated. The effect of each variable was evaluated using a separate regression model. The variables of proxy used were categorical, with the categories being “child's actual language,” “performance in clinical markers,” and “using both actual language and performance in clinical markers.” Test administrator was also a categorical variable with the categories being “parent” and “trained-examiners.” The variable of screening-diagnosis interval was dichotomously defined—intervals within 6 months were categorized as evaluating concurrent validity, whereas intervals of more than 6 months were categorized as evaluating predictive validity. The variable of screening age was also dichotomously defined with age 4 as the cut-off– those screened for children under the age of 4yo and those for children above 4yo. This categorization was primarily based on the age range of the sample, or the target screening age reported by the authors. Studies with age range that span across age 4 were excluded from the analysis. Considering the different thresholds used across studies and the correlated nature of sensitivity and specificity, meta-regression was conducted using a bivariate random effect model based on Reitsma et al. (22).

For studies examining multiple index tests and/or multiple cut-offs using the same population, only one screening test per category per study was included in the HSROC and meta-regression models. The test or cut-off with the highest Youden's index was included in the meta-analytical models. Youden's Index, *J*, was defined as


$$\begin{array}{l} J=Sensitivity+Specificity-1 \end{array}$$

All data analyses were conducted with RStudio Version 1.4.1106 using the package *mada* (24). Sensitivity analysis was carried out to exclude studies with a very high ROB (with 2 or more indicating a high risk in rating) to assess its influence on the results.

## Results

**Figure 1:**
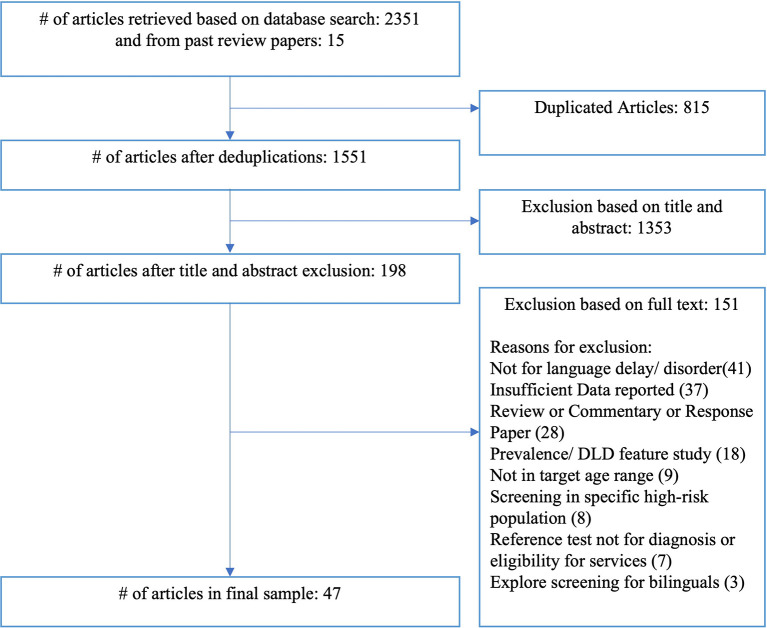
Flow-chart for the inclusion and exclusion of articles in literature search.

A total of 2351 articles, including 815 duplicates, were located using the search strategies, and an additional 15 articles were identified from previous review articles. After the inclusion and exclusion criteria were applied, a final sample of 47 studies were identified for inclusion in the review. Figure 1 shows the number of articles included and excluded at each stage of the literature search.

### Risk of Bias

**Figure 2:**
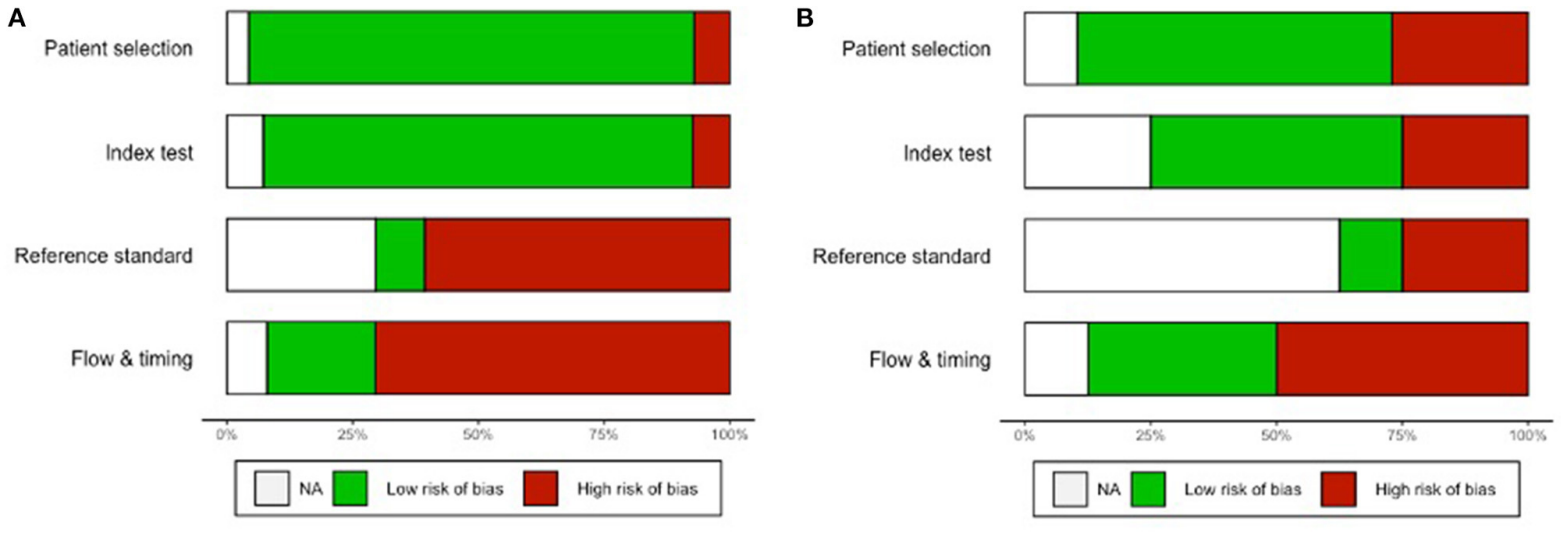
**(A)** Weighted and **(B)** unweighted overall risk-of-bias as assessed using QUADAS-2.

The weighted overall ROB assessment for the 47 studies is shown in Figure 2A, and the individual rating for each study is shown in Appendix B. Overall, half of the data was exposed to a high ROB in the administration and interpretation of the reference standard test, while almost two-thirds of the data had a high ROB in the flow and timing of the study. As indicated by the unweighted overall ROB summary plot in Figure 2B, half of the 47 studies were unclear about whether the administration and interpretation of the reference standard test would introduce bias. This was mainly attributable to a lack of reporting of the reference standard test performance. About half of the studies had a high ROB in the flow and timing of the study. This usually arose from a highly variable or lengthy follow-up period.

### Types and Characteristics of Current Screening Tools for Language Disorder

A total of 67 different index tests (or indices) were evaluated in the 47 included articles. The tests were either individual tests *per se* or part of a larger developmental test. The majority (50/67, 74.6%) of the screening tools examined children's actual language. Thirty of these index tests involved parents or caregivers as the main informants. Some of these screening tools were in the form of a questionnaire with Yes-No questions regarding children's prelinguistic skills, receptive language, or expressive language based on parent's observations. Some used a vocabulary checklist (e.g., CDI, LDS) in which parents checked off the vocabulary their child can was able to comprehend and/or produce. Some tools also asked parents to report their child's longest utterances according to their observation and generated indices. The other 20 index tests on language areas were administered by trained examiners such as nurses, pediatricians, health visitors or speech language pathologists (SLPs). These screening tools were constructed as checklists, observational evaluations, or direct assessments, tapping into children's developmental milestones, their word combinations and/or their comprehension, expression, and/or articulation. Some of these direct assessments involved the use of objects or pictures as testing stimuli for children.

A small proportion (3/67, 4.48%) of tests evaluated clinical markers performance including non-word repetitions and sentence repetitions rather than children's actual structural language skills or communication skills. About nine percent (6/67, 8.96%) screened for both language abilities and clinical markers. Both types of tests required trained examiners to administer them. The tests usually made use of a sentence repetition task and one test also included non-word repetition. Another nine percent (6/67, 8.96%) utilized indices from language sampling, such as percentage of grammatical utterances (PGU), mean length of utterances in words (MLU3-W), and number of different words (NDW) as proxies. These indices represented a child's syntactic, semantic, or morphological performance. The smallest proportion (2/67, 2.99%) of the tests elicited parental concerns about their children being screened for language disorder. One asked parents to rate their concern using a visual analog scale, while the other involved interviews with the parents by a trained examiner.

Sixty-five of the 67 screening tools had reported concurrent validity. Tables 1–5 summarize the characteristics of these 65 studies by the proxy used. Nine studies investigated the predictive validity of screening tools. Table 6 summarizes the studies. All the studies used child's actual language ability as the proxy.

**Table 1:** Studies involving tools based on a child's actual language ability.

**References**	**Agent**	**Index test**	**Reference standard test(s)**	**Sc. age (months)^**a**^**	***N***	**SN**	**SP**	**Accuracy^**b**^**	**Included in meta-analysis**
Allen and Bliss (25)	Trained personnel	The Northwestern Syntax Screening Test (26)	Sequenced inventory of communication development (27)	36–47	182	0.92	0.48	Below fair	✓
Blaxley et al. (28)	Trained personnel	Bankson Language Screening Test (29)	Developmental sentence scoring (30)	48–72	90	0.46	0.94	Below fair	✓
Burden et al. (31)	Parents/caregivers	The Parent Language Checklist and The Developmental Profile II (32)	Action Picture Test (33), Bus Story test (34), self-developed test on receptive and phonological ability	36–39	425	0.87	0.45	Below fair	✓
Carscadden et al. (35)	Parents/caregivers	Speech and Language Pathology Early Screening Instrument (35)	Receptive Expressive Emergent Language Test – 3^rd^ Edition (36)	17–23	53	0.91	0.95	Good	✓
Chaffee et al. (37)	Parents/caregivers	Minnesota Child Development Inventory – Comprehension Conceptual Language	Reynell Developmental Language Scales – revised (38)	24–87 M = 49	152	0.76	0.63	Below fair	✓
		Minnesota Child Development Inventory – Expressive Language (39)				0.89	0.45	Below fair	×
Dias et al. (40)	Parents/caregivers	Screening Tool by ASHA (41)	ABFW test (42)	0–60	962	0.83	0.99	Fair	✓
Dixon et al. (43)	Trained personnel	The Hackney Early Language Screening Test (43)	Reynell Developmental Language Scales (44), Lowe and Costello Symbolic Play Test (45)	30	40	0.94	0.95	Good	✓
Gray et al. (46)	Trained personnel	Expressive One-word Picture Vocabulary Test (47)	Referred by speech-language pathologist	48–60	62	0.71	0.71	Below fair	×
		Peabody Picture Vocabulary Test – III (48)				0.74	0.71	Below fair	×
		Receptive One-word Picture Vocabulary Test (49)				0.77	0.77	Below fair	✓
		Expressive Vocabulary Test (50)				0.71	0.68	Below fair	×
Guiberson (14)	Parents/caregivers	Parent reported vocabulary	Bilingual early childhood assessment team identification, parent report of concern, Spanish Preschool Language Scale – 4^th^ Edition (51)	24–35	62	0.86	0.88	Fair	✓
		Parent report of mean length of child's three longest utterances				0.46	0.93	Below fair	×
Guiberson and Rodriguez (52)	Parents/caregivers	Pilot Inventories III, translated version of MacArthur- Bates Communicative Development Inventory-III (53)	Spanish Preschool Language Scale – 4^th^ Edition (51)	36–62 M = 45.5	48	0.82	0.81	Fair	✓
		Ages and Stages Questionnaire – communication subscales (54)				0.59	0.92	Below fair	×
Guiberson et al. (55)	Parents/caregivers	Reported children's three longest utterances	Parent concern, enrollment in speech-language intervention services, Spanish Preschool Language Scale – 4^th^ Edition (51)	24–35 M = 29.4	45	0.91	0.86	Fair	✓
		Ages and Stages Questionnaire – communication subscales (56)				0.56	0.95	Below fair	×
		The Inventarios del Desarrollo de Habilidades Communicatives Palabras u Enunciado (57)				0.87	0.86	Fair	×
Guiberson et al. (58)	Parents/caregivers	Vocabulary score	SLP assessment, parental concern, Spanish Preschool Language Scale – 4^th^ Edition (51)	37–69 M = 53.7	82	0.79	0.77	Below fair	✓
		Language questions				0.74	0.69	Below fair	×
Heilmann et al. (59)	Parents/caregivers	MacArthur- Bates Communicative Development Inventory – Words and Sentences (60)	Preschool Language Scale – 3^rd^ Edition (61), language sampling	24 M = 23.8	100	0.68	0.98	Below fair	✓
Klee et al. (62)	Parents/caregivers	The Language Development Survey (63)	Mullen Scales of Early Learning (64), language sampling, parent interview, direct observation	24–26 M=24.7	64	0.91	0.87	Fair	×^c^
Klee et al. (65)	Parents/caregivers	The Language Development Survey (63)	Mullen Scales of Early Learning (64), language sampling, language sampling, parent interview, direct observation	24–26 M = 24.7	64	0.91	0.96	Good	✓
Laing et al. (66)	Trained Personnel	Structured Screening Test	Reynell Developmental Language Scales – III (67)	30–36 M=32	282	0.66	0.89	Below fair	✓
Law (68)	Trained personnel	Structured Screening Test	Reynell Developmental Language Scales (2^nd^ revision) (44)	30	189	0.86	0.76	Below fair	✓
Levett and Muir (69)	Trained personnel	Levett-Muir Language Screening Test (69)	Reynell Developmental Language Scales (revised) (70), Goldman-Fristoe Test of Articulation (71), Language Assessment and Remediation Procedure (72)	34.9–39.6	42	1	1	Good	✓
Visser-Bochane et al. (73)	Parents/caregivers	Early Language Screen (73)	LLC (74), SLC (75), LLP (76), SWP, SSP (77), LS-CCS (78), CCC-PCS (79)	12–72	124	0.79	0.86	Below fair	✓
Visser-Bochane et al. (80)	Trained personnel	The Dutch well child language screening protocol (80)	SLC (75), SWP, SSP (77)	26	265	0.62	0.93	Below fair	✓
Mattsson et al. (81)	Parents/caregivers and trained personnel	Questionnaire and Direct Observation by nurse	Clinical Examination by SLP	28–32 M = 30	105	0.81	0.87	Fair	✓
McGinty (82)	Parents/caregivers and trained personnel	The Mayo Early Language Screening Test (83)	Reynell Developmental Language Scales (44), Edinburgh Articulation Test (84)	18–60	200	0.84	0.7	Below fair	✓
Nair et al. (85)	Trained personnel	The Language Evaluation Scale Trivandrum For 0–3 Years (85)	Receptive-Expressive Emergent Language Scale (86)	0–36	643	0.96	0.78	Below fair	✓
Nayeb et al. (87)	Trained personnel	Nurse screening	Clinical Examination by SLP	29–31	100	1	0.85	Fair	✓
Puglisi et al. (15)	Trained personnel	Screening for Identification of Oral Language Difficulties by Preschool Teachers (15)	Expressive Vocabulary Test (88), Test for Reception of Grammar Version 2 (89), The Brazilian Children's Test of Pseudoword Repetition (90),	51–65 M = 57	100	0.86	0.95	Fair	✓
Rescorla (63)	Parents/caregivers	The Language Development Survey (63)	Reynell Developmental Language Scales (38)	23.7–34.4 M = 25.9	81	0.76	0.89	Below fair	✓
Rescorla and Alley (91)	Parents/caregivers	The Language Development Survey (63)	Reynell Developmental Language Scales (44)	23.7–34.4 M = 25.9	66	0.89	0.77	Below fair	✓
Sachse and Von Suchodoletz (92)	Parents/caregivers	German version of the CDI, Toddler Form-2 (93)	Language Test for 2-Year-Old Children (94)	24–26	117	0.93	0.87	Fair	✓
Stokes (95)	Trained personnel	Nurse screen	Language sampling, Reynell Developmental Language Scales (70)	34–40	366	0.77	0.97	Below fair	✓
	Parent/caregivers	Parent Questionnaire				0.75	0.95	Below fair	×
van Agt et al. (96)	Parents/caregivers	Van Wiechen (96)	Specialists' judgement	26–58 M = 39	8,877	0.71	0.89	Below fair	✓
		General Language Screen (97)				0.81	0.78	Below fair	×
		Language Screening Instrument – Parent Form (98)				0.86	0.73	Below fair	×
	Trained personnel	Language Screening Instrument – Child Test (98)				0.54	0.88	Below fair	×
Walker et al. (99)	Parents/caregivers	Early Language Milestone Scale (100)	Sequenced Inventory of Communication Development (27)	0–36	77	0.77	0.85	Below fair	✓
Wetherby et al. (101)	Parents/caregivers	Communication And Symbolic Behavior Scales – Developmental Profile, Infant-Toddler Checklist (102)	Behavior Sample	12–24 M = 14.5	151	0.89	0.74	Below fair	✓

*For tests that were validated against multiple cut-offs, only the one with highest Youden's index was shown; Sc. Age, screening age; MA, Meta-analysis; ASHA, American Speech-Language and Hearing Association; ABFW, Andrade CRF, Befi-Lopes DM, Fernandes FDM, Wertzner HF. Teste de Language Infantil nas Áreas de Fonologia, Vocabulário, Fluência e Pragmática. 2^nd^ ed. Barueri: Pró-Fono, 2011; LLC, Lexilist Comprehension; SLC, Schlichting test for Language Comprehension; LLP, Lexilist Production; SWP, Schlichting test for Word Production; SSP, Schlichting test for Sentence Production; LS-CCS – Language Standard – Communication Schlichting test for Language Composite Score; CCC-PCS, CCC-2-NL-Pragmatic Composite Score*.

a *Age of screening is reported in range or mean in the form of X_1_-X_2_ and M=X_3_; In case range or mean is not reported, the intended age for screening of the tool will be reports as X_4_*.

b *Based on Plante and Vance (19), Fair = over 0.8 in both sensitivity and specificity; Good = over 0.9 in both sensitivity and specificity*.

c *Not included because the sample was identical to Klee et al. (65)*.

**Table 2:** Studies involving tools based on clinical marker.

**References**	**Agent**	**Index test**	**Reference standard test(s)**	**Sc. age^**a**^ (months)**	***N***	**SN**	**SP**	**Accuracy^**b**^**	**Included in meta-analysis**
Guiberson et al. (58)	Trained personnel	Non-word Repetition	SLP assessment, parental concern, Spanish Preschool Language Scale – 4^th^ Edition (51)	37–69 M = 53.7	82	0.74	0.75	Below fair	✓
Kapalkova et al. (103)	Trained personnel	Non-word repetition	Clinical judgment and qualitative assessment	51–66	32	0.94	1	Good	✓
Nash et al. (104)	Trained personnel	The Grammar and Phonology Screening (GAPS) Test (105)	Clinical Evaluation of Language Fundamentals – Preschool, 2^nd^ Edition (106)	36–72 M = 62.3	106	0.3	0.91	Below fair	✓
Sturner et al. (107)	Trained personnel	The Sentence Repetition Screening Task (108)	Illinois Test of Psycholinguistic Abilities (109), Bankson Language Screening Test (29)	54–66 Med = 60	323	0.62	0.91	Below fair	✓
van der Lely et al. (110)	Trained personnel	The Grammar and Phonology Screening (GAPS) Test (105)	Assessment by SLP and educational psychologist	43–80	41	1	1	Good	✓

*For tests that were validated against multiple cut-offs, only the one with highest Youden's index was shown; Sc. Age, screening age*.

a *Age of screening is reported in range, mean or median in the form of X_1_-X_2_, M=X_3_ and Med=X_4_, respectively*.

b *Based on Plante and Vance (19), Fair = over 0.8 in both sensitivity and specificity; Good = over 0.9 in both sensitivity and specificity*.

**Table 3:** Studies involving tools based on both language ability and clinical marker.

**Study**	**Agent**	**Index test**	**Reference standard test(s)**	**Sc. age^**a**^ (months)**	***N***	**SN**	**SP**	**Accuracy^**b**^**	**Included in meta-analysis**
Allen and Bliss (25)	Trained personnel	The Fluharty Preschool Screening Test (111)	Sequenced Inventory of Communication Development (112)	36–47	182	0.6	0.81	Below fair	✓
Benavides et al. (113)	Trained personnel	Tamiz de Problemas de Lenguaje (113)	Clinical Evaluation of Language Fundamentals- 5^th^ edition, Spanish Version (114)	48–72	200	0.94	0.92	Good	✓
Blaxley et al. (28)	Trained personnel	The Fluharty Preschool Screening Test (115)	Developmental Sentence Scoring (116)	48–72	90	0.36	0.96	Below fair	✓
Bliss and Allen (117)	Trained personnel	The Screening Kit of Language Development (118)	Sequenced Inventory of Communication Development (112), clinical judgment by SLP	30–48	100	1	0.93	Good	✓
Lavesson et al. (119)	Trained personnel	Language tasks and non-word repetition (119)	SLP judgment based on test results	46–53 M = 48.5	328	0.84	0.96	Fair	✓
Matov et al. (120)	Trained personnel	Short Language Measures (121)	Clinical Evaluation of Language Fundamentals-4 (122)	63.6	126	0.94	0.93	Good	✓
Wright and Levin (123)	Trained personnel	Preschool Articulation and Language Screening (123)	SLP judgement based on test results	26–81	152	0.71	0.94	Below fair	✓

*For tests that were validated against multiple cut-offs, only the one with highest Youden's index was shown; Sc. Age, screening age*.

a *Age of screening is reported in range or mean in the form of X_1_-X_2_ and M=X_3_; In case range or mean is not reported, the intended age for screening of the tool will be reports as X_4._*.

b *Based on Plante and Vance (19), Fair = over 0.8 in both sensitivity and specificity; Good = over 0.9 in both sensitivity and specificity*.

**Table 4:** Studies involving tools based on language sampling.

**References**	**Agent**	**Index test**	**Reference standard test(s)**	**Sc. age^**a**^ (months)**	***N***	**SN**	**SP**	**Accuracy^**b**^**	**Included in meta-analysis**
Eisenberg and Guo (124)	Trained personnel	Percentage Grammatical Utterances	LI2: Previously diagnosed LI3: Parent rating, Structured Photographic Expressive Language Test – Preschool 2^nd^ Edition (125)	36–47	34	1	0.88	Fair	✓
		Percentage Sentence Point			34	1	0.82	Fair	×
		Percentage Verb Tense Usage (126)			34	1	0.82	Fair	×
Guiberson et al. (58)	Trained personnel	Ungrammaticality Index	SLP assessment, parental concern, Spanish Preschool Language Scale – 4^th^ Edition (51)	37–69 M = 53.7	82	0.59	0.67	Below fair	×
	Trained personnel	Mean Length of Utterances in Words				0.65	0.92	Below fair	✓
Guiberson (14)	Parents/caregivers	Number of Different Words	Bilingual early childhood assessment team identification, parent report of concern, Spanish Preschool Language Scale – 4^th^ Edition (51)	24–35	62	0.73	0.83	Below fair	✓

*For tests that were validated against multiple cut-offs, only the one with highest Youden's index was shown; Sc. Age, screening age; LI2, language impairment at age 2; LI3, language impairment at age 3*.

a *Age of screening is reported in range or mean in the form of X_1_-X_2_ and M=X_3_; In case range or mean is not reported, the intended age for screening of the tool will be reports as X_4_*.

b *Based on Plante and Vance (19), Fair = over 0.8 in both sensitivity and specificity; Good = over 0.9 in both sensitivity and specificity*.

**Table 5:** Studies involving tools based on parental concern.

**References**	**Agent**	**Index test**	**Reference standard test(s)**	**Sc. age^**a**^ (months)**	***N***	**SN**	**SP**	**Accuracy^**b**^**	**Included in meta-analysis**
Laing et al. (66)	Parents/caregivers	Parent led method	Reynell Developmental Language Scales – III (67)	30–36 M = 32	176	0.79	0.74	Below fair	✓
van Agt et al. (96)	Parents/caregivers	Visual analog scale to evaluate child's language development	Specialists' judgement	26–58 M = 39	8,877	0.76	0.81	Below fair	✓

*For tests that were validated against multiple cut-offs, only the one with highest Youden's index was shown; Sc. Age, screening age*.

a *Age of screening is reported in range or mean in the form of X_1_-X_2_ and M=X_3_; In case range or mean is not reported, the intended age for screening of the tool will be reports as X_4_*.

b *Based on Plante and Vance (19), Fair = over 0.8 in both sensitivity and specificity; Good = over 0.9 in both sensitivity and specificity*.

**Table 6:** Studies assessing predictive validity of screening tools.

**References**	**Agent**	**Index test**	**Sc. age** **(months)**	**Sc-V int. (months)**	**F/U age (months)**	**Reference standard test(s)**	***N***	**SN**	**SP**	**Accuracy^**a**^**	**MA included**
Bruce et al. (127)	Parents/caregivers and trained personnel	Direct assessment through play and parent questionnaire	18–22	NA	54	NELLI (128)^b^, The Test for Reception of Grammar (129)	43	0.6	0.85	Below fair	✓
Frisk et al. (130)	Trained personnel	Early Screening Profiles (131)	54	NA	60	Preschool Language Scale – 4^th^ Edition (132)	110	0.86	0.81	Fair	✓
	Parents/caregivers	Ages and Stages Questionnaire (54)				Bracken Basic Concepts Scale	110	0.84	0.66	Below fair	×
	Trained personnel	Battelle Developmental Inventory Screening Test (133)				Preschool (134) Language Scale – 4^th^ Edition (132)	110	0.68	0.86	Below fair	×
	Trained personnel	Brigance Preschool Screen (135)				Preschool Language Scale – 4^th^ Edition (132)	110	0.91	0.78	Below fair	×
Jessup et al. (136)	Trained personnel	Kindergarten Development Check (137)	48–54	8–12	NA	Clinical Evaluation of Language Fundamentals-4 (122)	286	0.5	0.93	Below fair	✓
Klee et al. (62)	Parents/caregivers	The Language Development Survey (63)	24	NA	36–40	Mullen Scales of Early Learning (64), language sampling, parent interview, direct observation	36	0.67	0.9	Below fair	✓
Pesco and O'Neill (138)	Parents/caregivers	Language Use Inventory (139)	24–47	14.54–54.76	NA	DELV- NR (140) CELF-2 (141), Children's Communication Checklist – 2^nd^ Edition (142)	236	0.81	0.93	Fair	✓
Sachse and Von Suchodoletz (92)	Parent/caregivers	German Version of The CDI, Toddler Form-2 (93)	24–26	12	NA	Language Test For 3–5-Year-Old Children (94)	102	0.94	0.61	Below Fair	×
	Trained personnel	Language Test for 2-Year-Old Children (94)	24–26	12	NA	Language Test For 3–5-Year-Old Children (94)	102	0.94	0.64	Below Fair	✓
Visser-Bochane et al. (80)	Trained personnel	The Dutch well-child language screening protocol (80)	*M* = 26	12	NA	SLC (75), SWP, SSP (77)	123	0.82	0.74	Below Fair	✓
Westerlund et al. (143)	Parents/caregivers	The Swedish Communication Screening at 18 Months of Age (144, 145)	18	NA	36	LO-3 (146, 147)	891	0.5	0.9	Below Fair	✓
	Trained personnel	Traditional Methods	18	NA	36	LO-3 (146, 147)	1,189	0.32	0.91	Below Fair	×
Wetherby et al. (101)	Parents/caregivers	Communication And Symbolic Behavior Scales – Developmental Profile Infant-Toddler Checklist (102)	12–24	*M* = 14.5	NA	Mullen Scales of Early Learning (148), Preschool Language Scale – 3^rd^ Edition (61)	246	0.81	0.79	Below Fair	×
	Trained personnel	Behavioral Sample	12–24	*M* = 18.2	NA	Mullen Scales Of Early Learning (148), Preschool Language Scale – 3^rd^ Edition (61)	90	0.84	0.85	Fair	✓

*For tests that were validated against multiple cut-offs, only the one with highest Youden's index was shown; Sc. Age, screening age; Sc-V int., Screening-validation Interval; F/U age, age at follow-up; DELV-NR, Diagnostic Evaluation of Language Variation – Norm Referenced; CELF-2, Clinical Evaluation of Language Fundamentals – Preschool, 2^nd^ Edition; LO-3, Language Observation at 3 years of age*.

a *Based on Plante and Vance (19), Fair = over 0.8 in both sensitivity and specificity; Good = over 0.9 in both sensitivity and specificity*.

b *Spraklig snabbscreening av forskolebarn 3–6 arunderlag for diagnostisering av art och grad av sprakstorning, Stora Fonemtestet. Pedagogisk, Grammatiktest. Pedagogisk*.

*^c^Based on Table 5 in the paper, description in the discussion differed from the figures in the table*.

### Screening Accuracy

Two of the 67 screening tools only reported predictive validity. Of the 65 screening tools that reported concurrent validity, about one-third (23/65, 35.4%) achieved at least fair accuracy and a smaller proportion (9/65, 13.8%) achieved good accuracy. The nine tools which achieved good accuracy include (i) Non-word Repetition, (ii) Speech and Language Pathology Early Screening Instrument (35), (iii) The Hackney Early Language Screening Test (43), (iv) The Language Development Survey (63), (v) Levett-Muir Language Screening Test (69), (vi) The Grammar and Phonology Screening (GAPS) Test (105), (vii) Tamiz de Problemas de Lenguaje (113), (viii) The Screening Kit of Language Development (117) and (ix) Short Language Measures (120).

#### Screening Performance by Proxy and Screening-Diagnosis Interval

Screening tools based on children's actual language ability had a sensitivity ranging from 0.46 to 1 (median = 0.81) and a specificity of 0.45 to 1 (median = 0.86). About 30% of the studies showed that their tools achieved at least fair accuracy, while 8.89% achieved good accuracy. Screening tools using clinical markers had a sensitivity ranging from 0.3 to 1 (median = 0.71) and a specificity of 0.45 to 1 (median = 0.91). Two of the five studies^1^ (40%) evaluating screening tools based on clinical markers showed their tools had good sensitivity and good specificity, but the other three studies showed a sensitivity and a specificity below fair. Concerning screening tools based on both actual language ability and clinical marker performance, the sensitivity ranged from 0.36 to 1 (median = 0.84), and the specificity ranged from 0.81 to 0.96 (median=0.93) and above half of these studies (4/7^2^, 57.1%) achieved at least fair performance in both sensitivity and specificity, and 3 of the 7 studies achieved good performance. Screening tools based on indices from language sampling had sensitivity ranging from 0.59 to 1 (median = 0.865) and specificity ranging from 0.67 to 0.92 (median = 0.825). Half of these six screening tools achieved fair accuracy, but none achieved good accuracy. None of the two screening tools based on parental concern achieved at least fair screening accuracy.

Fifteen of the 65 studies also reported predictive validity, with a sensitivity ranging from 0.32 to 0.94 (median = 0.81) and a specificity ranging from 0.61 to 0.93 (median = 0.85). Three of the tools (20%) achieved at least fair accuracy in both sensitivity and specificity, but none of them were considered to have good accuracy.

#### Test Performance Based on HSROC

Three HSROC curves were generated for screening tools based on language ability, clinical markers, both language ability and clinical markers, and those assessing concurrent validity. Two HSROC curves were generated for screening tools administered by trained examiners and parents/ caregivers, respectively. Two HSROC curves were generated for screening under and above the age of 4, respectively. A separate HSROC curve was generated for screening tools assessing predictive validity. Screening based on indices from language sampling (*n* = 3) or parental concern (*n* = 2) were excluded from the HSROC analysis due to the small number of primary studies.

**Figure 3:**
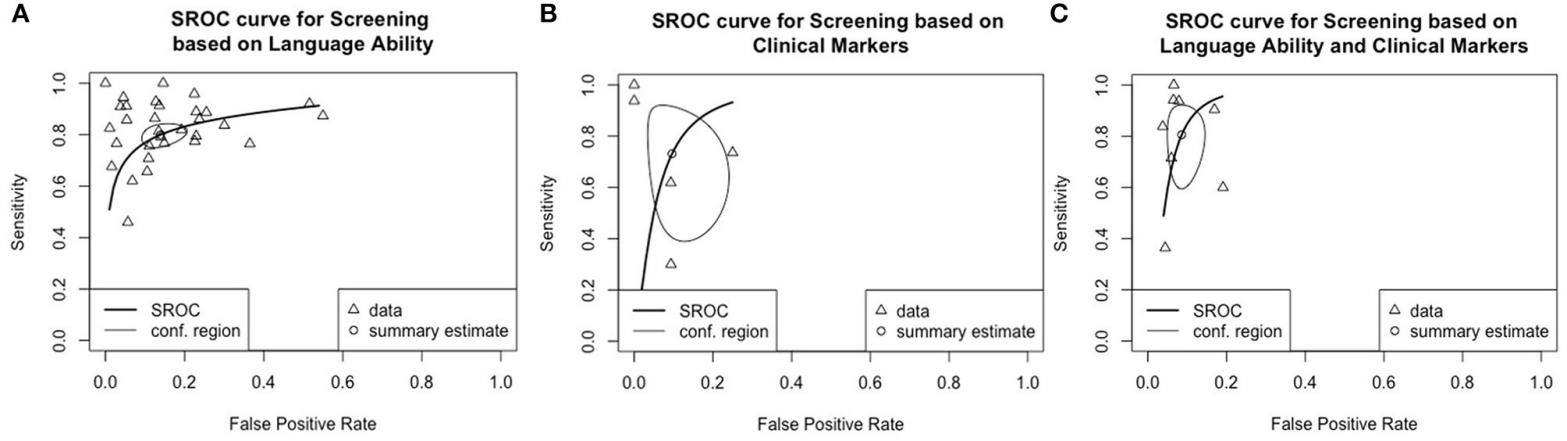
Summary receiver operating characteristics curves for screening tools based on **(A)**, language ability, **(B)** clinical markers, and **(C)** language & clinical markers.

**Figure 4:**
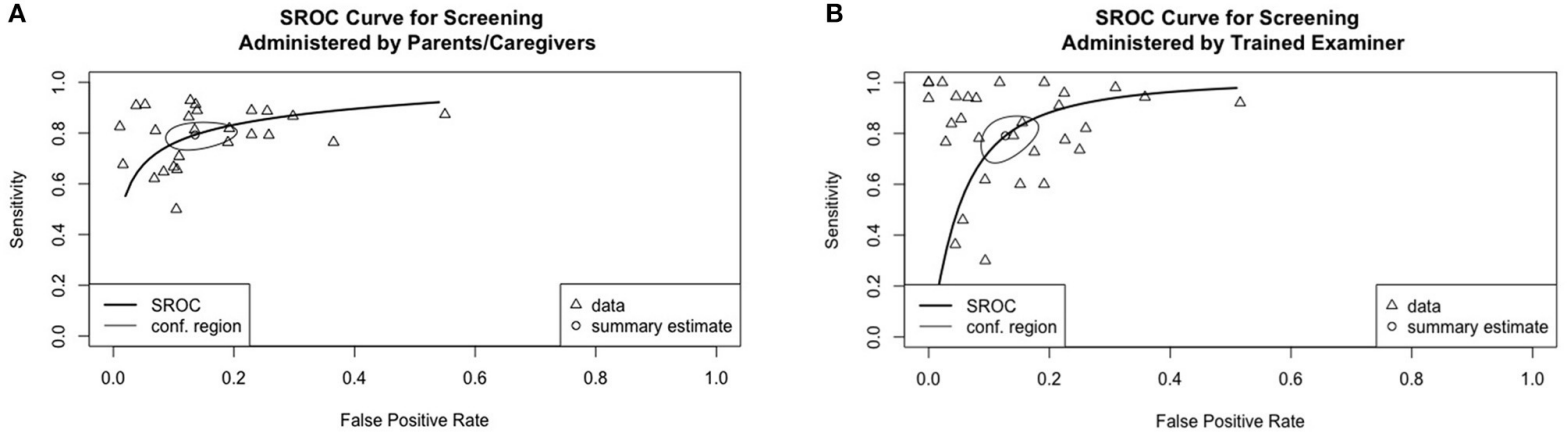
Summary receiver operating characteristics curves for screening tools administered by **(A)** parents/caregivers and **(B)** trained examiners.

**Figure 5:**
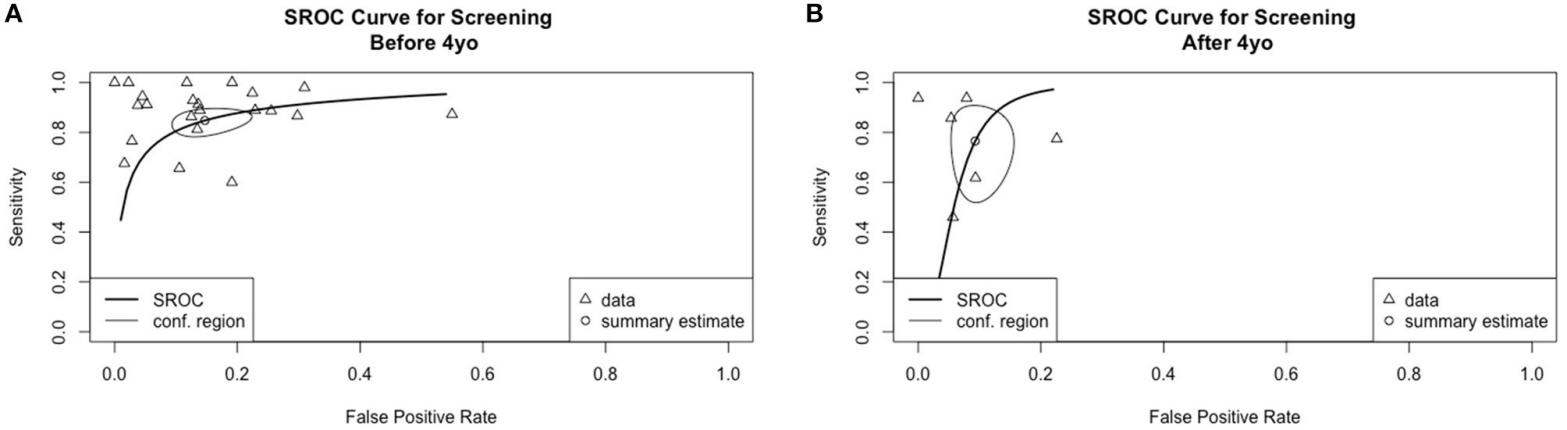
Summary receiver operating characteristics curves for screening **(A)** under 4-year-old and **(B)** above 4-year-old.

**Figure 6:**
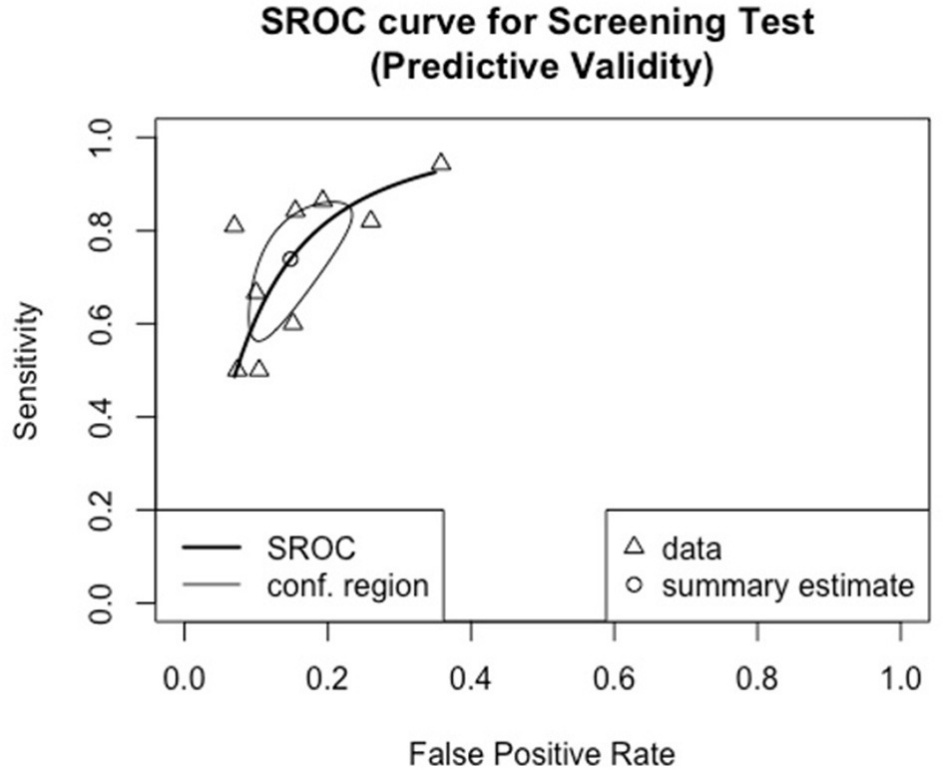
Summary receiver operating characteristic curve for screening tools reporting predictive validity.

Figure 3 shows the overall performance of screening tools based on language ability, clinical markers and both. Visual inspection of the plotted points and confidence region revealed considerable variation in accuracy in all three major types of screening tools. The summary estimates and confidence regions indicated that the overall performance of screening tools based on language ability achieved fair specificity (<0.2 in false positive rate) but fair-to-poor sensitivity. Screening tools based on clinical markers showed considerable variation in both sensitivity and specificity in that both measures ranged from good-to-poor. Screening tools based on both language ability and clinical markers achieved good-to-fair specificity, but fair-to-poor sensitivity. Figure 4 shows the overall performance of screening tools administered by parents/caregivers or trained examiners. Visual inspection revealed that both types of screening tools achieved fair-to-poor sensitivity and good-to-fair specificity. Figure 5 shows the overall performance of screening for children under and above 4yo, respectively. Visual inspection revealed screening under 4yo achieved good-to-poor sensitivity and specificity, while screening above 4yo achieved good-to-poor sensitivity and good-to-fair specificity. Figure 6 shows the performance of the screening tools evaluating predictive validity. These screening tools achieved fair-to-poor sensitivity and specificity.

### Meta-Regression Investigating Effects of Screening Proxy, Test Administrator, Screening-Diagnosis Interval, and Age of Screening

The effects of screening proxy, test administrator, screening-diagnosis interval and age of screening on screening accuracy were investigated using bivariate meta-regression. Table 7 summarizes the results. Screening tools with <6-month screening-diagnosis interval (i.e., concurrent validity) were associated with higher sensitivity when compared to those with longer than a 6-month interval (i.e., predictive validity). Tools using language ability as the proxy showed a marginally significantly higher sensitivity than those based on clinical markers. Screening tools based on language ability and those based on both language ability and clinical markers appeared to show a similar degree of sensitivity. For tools assessing concurrent validity, screening under the age of 4 had a higher sensitivity with marginal statistical significance but showed similar specificity with screening above the 4yo. As for tools assessing predictive validity, screening under and above 4yo appeared to show similar sensitivity and specificity. Similarly, screening tools relying on parent report and those conducted by trained examiners appeared to show a similar sensitivity. Despite the large variability in specificity, none of the factors in the meta-regression model explained this variability.

**Table 7:** Bivariate meta-regression on studies-related factors on sensitivity and false-positive rate.

**Factor**	**Transformed sensitivity**				**Transformed false positive rate**			
	**Coeff**.	**95% CI**		***p*-value**	**Coeff**.	**95% CI**		***p*-value**
		**LL**	**UL**			**LL**	**UL**	
Types (L vs. Cm)	0.657	−0.055	1.370	0.070^#^	0.325	−0.774	1.423	0.562
Types (L vs. Mx)	−0.300	−0.855	0.255	0.290	0.435	−0.330	1.201	0.265
Types (Mx vs. Cm)	0.885	−0.244	2.015	0.124	−0.094	−0.958	0.770	0.832
Time (P vs. C)	−0.528	−1.018	−0.037	0.035^*^	−0.016	−0.726	0.695	0.965
Sc. AgeC (<4yo vs. ≥4*yo*)	1.676	−0.115	1.467	0.094^#^	0.560	−0.292	1.412	0.198
Sc. AgeP (<4yo vs. ≥4*yo*)	1.061	−1.115	3.238	0.339	0.663	−0.737	2.064	0.353
Informant (TP∧ vs. Pa)	−0.003	−0.525	0.519	0.992	−0.031	−0.836	0.773	0.939

*First group in the bracket as the reference; L, language only; Cm, clinical markers; Mx, both language and clinical markers; P, predictive validity; C, concurrent validity; Pa, parent; TE, trained personnel; ScAgeC, Screening Age (for studies evaluating concurrent validity); ScAgeC, Screening Age (for studies evaluating predictive validity)*.

# *p < 0.1*;

* *p < 0.05*.

Results of sensitivity analysis after excluding studies with high ROB are illustrated in Table 8. The observed higher sensitivity for screening tools using actual language as proxy compared with those using clinical markers became statistically significant. The difference in sensitivity between screening tools assessing concurrent validity and those assessing predictive validity appeared to be larger than before the removal of the high ROB studies. However, the observed marginal difference between screening under and above 4yo became non-significant after the exclusion of high-risk studies. Similar to the results without excluding studies with high ROB, none of the included factors in sensitivity analysis explained variation in specificity.

**Table 8:** Bivariate meta-regression of study-related factors on sensitivity and false-positive rate excluding high ROB studies.

**Factor**	**Transformed sensitivity**				**Transformed false positive rate**			
	**Coeff**.	**95% CI**		***p*-value**	**Coeff**.	**95% CI**		***p*-value**
		**LL**	**UL**			**LL**	**UL**	
Types (L∧ vs. Cm)	0.960	0.291	1.629	0.005**	−0.020	−1.295	1.256	0.976
Types (L∧ vs. Mx)	−0.173	−0.784	0.439	0.580	0.157	−0.753	1.067	0.735
Types (Mx∧ vs. Cm)^a^	-	-	-	-	-	-	-	-
Time (P∧ vs. C)	−0.819	−1.377	−0.262	0.004^*^	−0.104	−1.009	0.801	0.822
Sc. Age C(<4yo vs. ≥4*yo*)	0.234	−0.926	1.394	0.692	0.520	−0.388	1.428	0.262
Sc. Age P(<4yo vs. ≥4*yo*)^a^	-	-	-	-	-	-	-	-
Informant (TE∧ vs. Pa)	0.149	−0.514	0.812	0.660	0.160	−0.870	1.189	0.761

*First group in the bracket as the reference; L, language only; Cm, clinical markers; Mx, both language and clinical markers; P, predictive validity; C, concurrent validity; Pa, parent; TE, trained examiner; ScAgeC, Screening Age (for studies evaluating concurrent validity); ScAgeC, Screening Age (for studies evaluating predictive validity)*.

a *Too few studies after exclusion for a valid analysis*.

*^#^p < 0.1*.

* *p < 0.05*.

## Discussion

The present review shows that currently available screening tools for language disorders during preschool years varies widely in their design and screening performance. Large variability in screening accuracy across different tools was a major issue in screening for language disorder. The present review also revealed that the variations arose from the choices of proxy and screening-diagnosis interval.

Screening tools based on children's actual language ability were shown to have higher sensitivity than tools based on clinical markers. The fact that screening tools based on clinical markers did not prove to be sensitive may be related to the mixed findings from primary studies. Notably, one of the primary studies using non-word repetition and sentence repetition tasks showed perfect accuracy in classifying all children with and without language disorder (110). The findings, however, could not be replicated in another study, using exactly the same test, which identified only 3 of the 10 children with language disorder (104). The difference highlighted the large variability in the performance of non-word and sentence repetition even among children with language disorders, in addition to the inconsistent difference found between children with and without language disorder (149). Another plausible explanation for the relatively higher sensitivity of using child's actual language skills lies in the resemblance between the items used for screening based on the child's actual language and the diagnostic tests used as the reference standard. Differences in task design and test item selection across studies may have further increased the inconsistencies (149). Therefore, in future tool development or refinement, great care should be taken in the choice of screening proxy. More systematic studies directly comparing how different proxies and factors affect screening accuracy are warranted.

There was no evidence that other factors related to tool design, such as the test administrators of the screening tools, explained variability in accuracy. In line with a previous review (13), parent-report screening appeared to perform similarly to screening administered by trained examiners. This seemingly comparable accuracy supports parent-report instruments as a viable tool for screening, in addition to their apparent advantage of lower cost of administration. Primary studies directly comparing both types of screening in the same population may provide stronger evidence concerning the choice of administrators.

As predicted, long term prediction was harder to achieve than estimating concurrent status. Meta-analysis revealed that screening tools reporting predictive validity showed a significantly lower sensitivity than that of tools reporting concurrent validity, which was also speculated in the previous review (13). One possible explanation lies in the diverse developmental trajectories of language development in the preschool years. Some of the children who perform poorly in early screening may recover spontaneously at a later time point, while some who appeared to be on the right track at the beginning may develop language difficulties later on (7). Current screening tools might not be able to capture this dynamic change in language development in the preschool years, resulting in lower predictive validity than expected. Hence, language disorder screening should concentrate on identifying or introducing new proxies or metrics that are sensitive to the dynamic nature of language development. Vocabulary *growth* estimates, for example, might be more sensitive to long-term outcomes than a single point estimation (150). Although the current review has shown that different proxies has been used in screening language disorder, there is a limited number of studies examining how proxies other than children's actual language ability perform in terms of predictive validity. It would be useful to investigate the interaction between the proxy used and the screening-diagnosis interval in future studies.

Age of screening was expected to be affected by the varying developmental trajectories. Screening at an earlier age might have lower accuracy than screening at a later age when language development becomes more stable. This expected difference was not found in the current meta-analysis. However, it is worth noting that screening tools used at different ages not only differed in the age of screening, but also other domains. In the meta-analysis, over half (55%, 16/29) of the screening under 4 relied on parent reports and used tools such as vocabulary checklists and reported utterances while none of the screening above 4 (0/8) were based on parent reports. Inquiry about the effect of screening age on screening accuracy is crucial as it has direct implication on the optimal time of screening. Future studies that compare the screening accuracy at different ages with the method of assessment being kept constant (e.g., using the same screening tool) may reveal a clearer picture.

Overall, only a small proportion of all the available screening tools achieved good accuracy in identifying both children with and without language disorder. Yet, there is still insufficient evidence to recommend any screening tool, especially given the presence of ROB in some studies. Besides, the limited number of valid tools may explain partly why screening for language disorder has not yet been adopted as a routine surveillance exercise in primary care, in that the use of any one type of screening tools may result in a considerable amount of over-identification and missing cases, which can lead to long term social consequences (19). As shown in the current review, in the future development of screening tools, the screening proxy should be carefully chosen in order to maximize test sensitivity. However, as tools that have good accuracy are limited, there remains room for discussion on whether future test development should aim at maximizing sensitivity even at the expense of specificity. The cost of over-identifying a false-positive child for a more in-depth assessment might be less than that of under-identifying a true-positive child and depriving the child of further follow-ups (104). If this is the case, the cut-off for test positivity can be adjusted. The more stringent the criteria used in screening, the higher the sensitivity the test yield but with the trade-off of a decrease in specificity. However, the decision should be made by fully acknowledging the harms and benefits, which has not been addressed in the current review. While an increase in sensitivity by adjusting the cut-off might lead to the benefit of better follow-ups, the accompanying increase in false positive rate might lead to the harms of stigmatization and unnecessary procedures. Given the highly variable developmental trajectories in asymptomatic children, another direction for future studies could be to evaluate the viability of targeted screening in a higher-risk population and compare it with universal screening.

This is the first study to use meta-analytical techniques specifically to evaluate the heterogeneity in screening accuracy of tools for identifying children with language disorder. Nonetheless, there were several limitations of the study. One limitation was related to the variability and validity of the gold standard in that the reference standard tests. Different countries or regions use different localized standardized or non-standardized tools and criteria to define language disorder. There is no one consensual or true gold standard. More importantly, the significance and sensitivity and specificity of the procedures used to identify children with language disorders in those reference tests were not examined. Some reference tests may employ arbitrary cut-offs (e.g.,−1.25 SD) to define language disorders while some researchers advocate children's well-being as the outcome, such that when children's lives are negatively impacted by their language skills, they are considered as having language disorders (151). This lack of consensus might further explain the diverse results or lack of agreement in replication studies.

Another limitation of the study was that nearly all the included studies had at least some ROB. This was mainly due to many unreported aspects in the studies. It is suggested that future validation studies on screening tools should follow reporting guidelines such as STARD (152). A third limitation was that the rating of ROB only involved one rater, and more raters may minimize potential bias. Lastly, not all included screening tools were analyzed in the meta-analysis. Some studies evaluated multiple screening tools at a number of cut-offs or times of assessment. Only one data point per study was included in the meta-analysis and the data used in meta-analysis were chosen based on Youden's index. This selection would inevitably inflate the accuracy shown in the meta-analysis. With the emergence of new methods for meta-analysis for diagnostic studies, more sophisticated methods for handling this complexity of data structure may be employed in future reviews.

This review shows that current screening tools for developmental language disorder vary largely in accuracy, with only some achieving good accuracy. Meta-analytical data identified some sources for heterogeneity. Future development of screening tools should aim at improving overall screening accuracy by carefully choosing the proxy or designing items for screening. More importantly, metrics that are more sensitive to persistent language disorder should be sought. To fully inform surveillance for early language development, future research in the field can also consider broader aspects, such as the harms and benefits of screening as there is still a dearth of evidence in this respect.

## Data Availability Statement

Publicly available datasets were analyzed in this study. This data can be found at: Reference lists of the article.

## Author Contributions

KS and CT conceived and designed the study, wrote the paper, conducted the format and tables, reviewed, and edited the manuscript. KS performed the statistical analysis. All authors have approved the final manuscript for submission.

## Conflict of Interest

The authors declare that the research was conducted in the absence of any commercial or financial relationships that could be construed as a potential conflict of interest.

## Publisher's Note

All claims expressed in this article are solely those of the authors and do not necessarily represent those of their affiliated organizations, or those of the publisher, the editors and the reviewers. Any product that may be evaluated in this article, or claim that may be made by its manufacturer, is not guaranteed or endorsed by the publisher.

## Footnotes

^1^The total number here refers to the number of studies: there were five studies evaluating tools based on clinical markers, but there were in total three different tests; hence number is different from that in types and characteristics of current screening tools for language disorder.

^2^The total number here refers to the number of studies: there were seven studies evaluating tools based on both actual language and clinical markers, but there were in total six different tests; hence number is different from that in types and characteristics of current screening tools for language disorder.
