# Corrigendum: Systematic review and meta-analysis of screening tools for language disorder

**DOI:** 10.3389/fped.2023.1288921

**Published:** 2023-09-22

**Authors:** Kevin K. H. So, Carol K. S. To

**Affiliations:** Academic Unit of Human Communication, Development, and Information Sciences, Faculty of Education, The University of Hong Kong, Hong Kong, Hong Kong SAR, China

**Keywords:** surveillance, screening, language disorder, PRISMA review, meta-analysis, summary receiver-operating characteristics, meta-regression

A Corrigendum on Systematic review and meta-analysis of screening tools for language disorder By So KKH and To CKS (2022). Front. Pediatr. 10:801220. doi: 10.3389/fped.2022.801220

In the published article, the name of one of the authors was incorrectly spelled in the reference 87, the author name has been changed from Lagerbere D. to Lagerberg D. The corrected reference appears below.

“Nayeb L, Lagerberg D, Westerlund M, Sarkadi A, Lucas S, Eriksson M. Modifying a language screening tool for three-year-old children identified severe language disorders six months earlier. *Acta Paediatrica*. (2019) 108:1642–8. doi: 10.1111/apa.14790”

The authors apologize for this error and state that this does not change the scientific conclusions of the article in any way. The original article has been updated.

