# Supplementary material for: Systematic Review and Meta-Analysis of Screening Tools for Language Disorder
Source: Front Pediatr. 2022 Feb 23;10:801220. doi: 10.3389/fped.2022.801220 (PMC8904415; doi:10.3389/fped.2022.801220)
Supplement: Supplementary file 1 [file Data_Sheet_1.docx]

**Appendix A**

**Table A1**

*Search Strategies*

| Database | Engine | Search field | Search strategy |
| --- | --- | --- | --- |
| CINAHL Plus | EBScoHost | TI+AB | TI ( ( child* OR preschool*) AND ("language disorder*" OR "language impairment*" OR "specific language disorder*" OR "language delay" ) AND ( screen*) ) OR AB ( ( child* OR preschool* ) AND ( "language disorder*" OR "language impairment*" OR "specific language disorder*" OR "language delay" ) AND ( screen*) ) |
| ComDisDome | ProQuest | TI+AB | ti(( child* OR preschool*) AND ( "language disorder*" OR "language impairment*" OR "specific language disorder*" OR "language delay" ) AND ( screen*) ) OR ab(( child* OR preschool*) AND ("language disorder*" OR "language impairment*" OR "specific language disorder*" OR "language delay" ) AND ( screen*) ) |
| PsycInfo, PsycArticles,  PsycTests | ProQuest | TI+AB | ti(( child* OR preschool*) AND ( "language disorder*" OR "language impairment*" OR "specific language disorder*" OR "language delay" ) AND ( screen*) ) OR ab(( child* OR preschool*) AND ("language disorder*" OR "language impairment*" OR "specific language disorder*" OR "language delay" ) AND ( screen*) ) |
| ERIC | EBScoHost | TI+AB | TI ( ( child* OR preschool*) AND ("language disorder*" OR "language impairment*" OR "specific language disorder*" OR "language delay" ) AND ( screen*) ) OR AB ( ( child* OR preschool*) AND ( "language disorder*" OR "language impairment*" OR "specific language disorder*" OR "language delay" ) AND ( screen*) ) |
| PubMed | PubMed | / | ((("Language Disorders"[Mesh] OR "Specific Language Disorder"[Mesh]  OR "Language Development Disorders"[Mesh] OR "Communication  Disorders"[Mesh]) AND "Mass Screening"[Mesh])) AND "Child,  Preschool"[Mesh] |
| Web of Science | / | Topic | TOPIC: (( child* OR preschool*) AND ("language disorder*" OR "language impairment*" OR "specific language disorder*" OR "language delay" ) AND ( screen*)) |
| Scopus | Scopus | TI+AB | TITLE-ABS ( ( child* OR preschool*) AND ( "language disorder*" OR "language impairment*" OR "specific language disorder*" OR "language AND delay" ) AND ( screen* ) ) |

**Table A2**

*Inclusion and Exclusion Criteria Used in Literature Search*

| Factor | Inclusion | Exclusion |
| --- | --- | --- |
| Study design | Cross-sectional, prosepctive | / |
| Research aim | Validating screening tools  against gold standard; comparing screening tools/ methods | Validating Diagnostic tools  Treatment studies  Review paper  Prevalence study  DLD feature study |
| Endpoint of screnning | Developmental language disorder, language delay,  language disorder | Other devleopmental disorder (e.g. fluency disorder) |
| Reference standard test | Language assessment/ enrollment in SLP services | Parental concern  Another Screening Tool |
| Time | Any | / |
| Agent | Parent / trained personnel | / |
| Ethnicity | All | / |
| Source | Peer-reviewed journal | Gray Literature, conference paper |
| Population | General or referral clinic | Disordered population |
| Data | Reported sensitivity, specificity and prevalence / TP, TN, FP, FN/ sample size | / |
| Language | English | Other language |
| Age | 6 or below |  |

**Appendix B**

**Figure B1**

*Traffic Light Chart for Individual Studies Based on QUADAS-2*

| Low Risk | 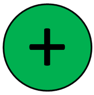 |
| --- | --- |
| High Risk | 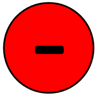 |
| Unclear | 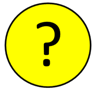 |

|  | D1 | D2 | D3 | D4 | Overall |
| --- | --- | --- | --- | --- | --- |
| Allen & Bliss, 1987 | 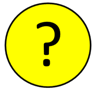 | 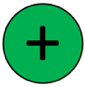 | 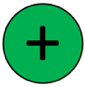 | 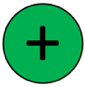 | 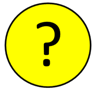 |
| Benavides *et al.* 2018 | 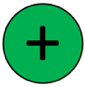 | 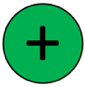 | 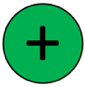 | 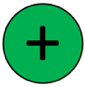 | 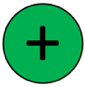 |
| Blaxley *et al.,* 1983 | 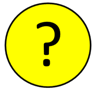 | 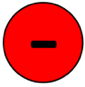 | 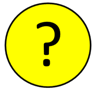 | 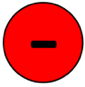 | 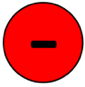 |
| Bliss & Allen, 1984 | 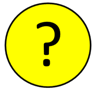 | 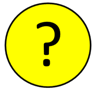 | 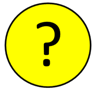 | 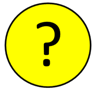 | 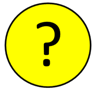 |
| Bruce *et al.* 2003 | 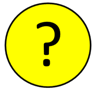 | 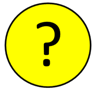 | 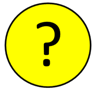 | 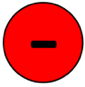 | 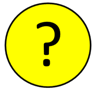 |
| Burden et al. 1996 | 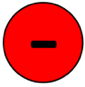 | 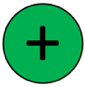 | 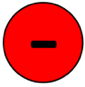 | 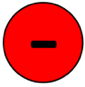 | 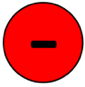 |
| Carscadden *et al.*, 2010 | 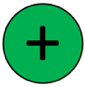 | 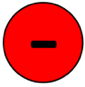 | 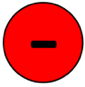 | 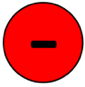 | 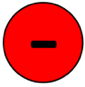 |
| Chaffee *et al.,* 1990 | 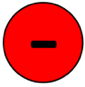 | 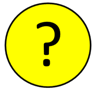 | 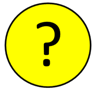 | 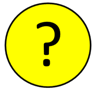 | 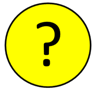 |
| Dias *et al.,* 2020 | 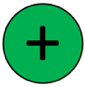 | 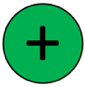 | 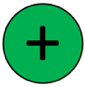 | 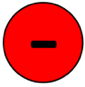 | 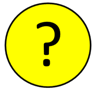 |
| Dixon *et al.,* 1988 | 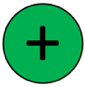 | 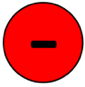 | 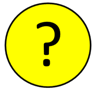 | 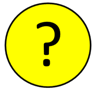 | 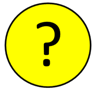 |
| Eisenberg & Guo, 2013 | 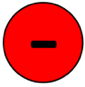 | 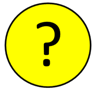 | 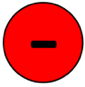 | 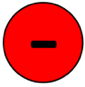 | 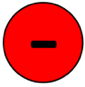 |
| Frisk *et al.*, 2009 | 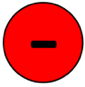 | 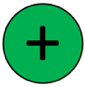 | 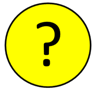 | 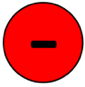 | 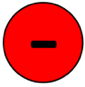 |
| Gray *et al.,* 1999 | 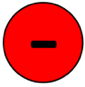 | 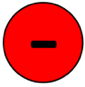 | 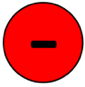 | 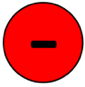 | 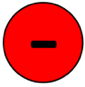 |
| Guiberson & Rodriguez, 2010 | 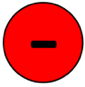 | 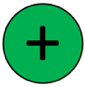 | 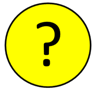 | 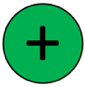 | 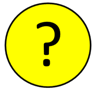 |
| Guiberson, 2016 | 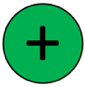 | 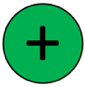 | 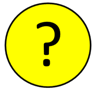 | 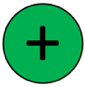 | 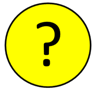 |
| Guiberson *et al.,* 2011 | 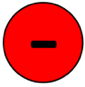 | 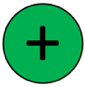 | 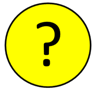 | 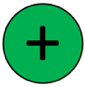 | 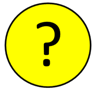 |
| Guiberson *et al.,* 2015 | 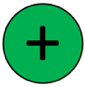 | 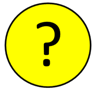 | 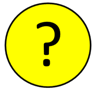 | 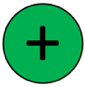 | 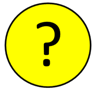 |
| Heilmann *et al.,* 2005 | 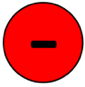 | 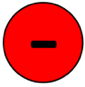 | 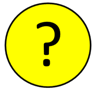 | 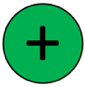 | 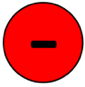 |
| Jessup *et al.,* 2008 | 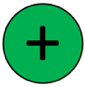 | 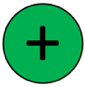 | 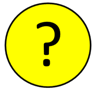 | 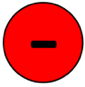 | 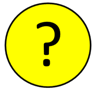 |
| Kapalkova *et al.,* 2013 | 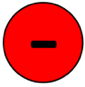 | 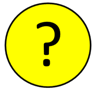 |  |  |  |
| Klee *et al.,* 1998 |  |  |  |  |  |
| Klee *et al.,* 2000 |  |  |  |  |  |
| Laing *et al.,* 2002 |  |  |  |  |  |
| Lavesson *et al.,* 2018 |  |  |  |  |  |
| Law, 1994 |  |  |  |  |  |
| Levett & Muir, 1983 |  |  |  |  |  |
| M. I. Visser-Bochane *et al.,* 2020 |  |  |  |  |  |
| M. Visser-Bochane *et al.,* 2020 |  |  |  |  |  |
| Matov *et al.,* 2020 |  |  |  |  |  |
| Mattsson *et al.,* 2001 |  |  |  |  |  |
| McGinty, 2000 |  |  |  |  |  |
| Nair *et al.,* 2013 |  |  |  |  |  |
| Nash *et al.,* 2011 |  |  |  |  |  |
| Nayeb *et al.,* 2019 |  |  |  |  |  |
| Pesco & O'Neill, 2012 |  |  |  |  |  |
| Puglisi *et al.,* 2020 |  |  |  |  |  |
| Rescorla & Alley, 2001 |  |  |  |  |  |
| Rescorla, 1989 |  |  |  |  |  |
| Sachse & Von Suchodoletz, 2008 |  |  |  |  |  |
| Stokes, 1997 |  |  |  |  |  |
| Sturner *et al.,* 1996 |  |  |  |  |  |
| van Agt *et al.,* 2007 |  |  |  |  |  |
| van der Lely *et al.,* 2011 |  |  |  |  |  |
| Walker *et al.,* 1989 |  |  |  |  |  |
| Westerlund *et al.,* 2006 |  |  |  |  |  |
| Wetherby *et al.,* 2003 |  |  |  |  |  |
| Wright *et al.,* 1971 |  |  |  |  |  |

*Note.*  Domains:

D1: Patient selection

D2: Administration and interpretation of index test

D3: Administration and interpretation of reference standard test

D4: Flow and timing

Overall: High risk defined as having 2 or more high risks D1 to D4 and low risk defined as having low risks in all D1-D4
